# Time- and Task-Dependent Non-Neural Effects of Real and Sham TMS

**DOI:** 10.1371/journal.pone.0073813

**Published:** 2013-09-05

**Authors:** Felix Duecker, Tom A. de Graaf, Christianne Jacobs, Alexander T. Sack

**Affiliations:** 1 Department of Cognitive Neuroscience, Faculty of Psychology and Neuroscience, Maastricht University, Maastricht, The Netherlands; 2 Maastricht Brain Imaging Center, Maastricht University, Maastricht, The Netherlands; Radboud University Nijmegen, Netherlands

## Abstract

Transcranial magnetic stimulation (TMS) is widely used in experimental brain research to manipulate brain activity in humans. Next to the intended neural effects, every TMS pulse produces a distinct clicking sound and sensation on the head which can also influence task performance. This necessitates careful consideration of control conditions in order to ensure that behavioral effects of interest can be attributed to the neural consequences of TMS and not to non-neural effects of a TMS pulse. Surprisingly, even though these non-neural effects of TMS are largely unknown, they are often assumed to be unspecific, i.e. not dependent on TMS parameters. This assumption is inherent to many control strategies in TMS research but has recently been challenged on empirical grounds. Here, we further develop the empirical basis of control strategies in TMS research. We investigated the time-dependence and task-dependence of the non-neural effects of TMS and compared real and sham TMS over vertex. Critically, we show that non-neural TMS effects depend on a complex interplay of these factors. Although TMS had no direct neural effects, both pre- and post-stimulus TMS time windows modulated task performance on both a sensory detection task and a cognitive angle judgment task. For the most part, these effects were quantitatively similar across tasks but effect sizes were clearly different. Moreover, the effects of real and sham TMS were almost identical with interesting exceptions that shed light on the relative contribution of auditory and somato-sensory aspects of a TMS pulse. Knowledge of such effects is of critical importance for the interpretation of TMS experiments and helps deciding what constitutes an appropriate control condition. Our results broaden the empirical basis of control strategies in TMS research and point at potential pitfalls that should be avoided.

## Introduction

Transcranial magnetic stimulation (TMS) is a non-invasive interference technique that is widely used in experimental brain research [1–4]. By exposing the brain to a rapidly changing magnetic field, TMS allows manipulation of brain activity in human volunteers during task execution with excellent temporal resolution and high spatial precision. Next to the intended neural effects of TMS, every TMS pulse produces a distinct clicking sound and sensations on the head. The presence of these non-neural effects creates a strong need for appropriate control conditions. TMS experiments have to be designed in such a way that behavioral effects of interest can be attributed to the neural consequences of TMS and not to non-neural effects of a TMS pulse.

Even though appropriate control conditions are key to the interpretation of any TMS effect, there is a surprising lack of empirical evidence on the non-neural effects of TMS. Only in the late 1990s, this issue briefly captured attention with some studies reporting intersensory facilitation when a TMS pulse is administered in close temporal proximity to a target stimulus [5–8]. Since then, the TMS community has rarely addressed the non-neural effects of TMS explicitly and rather developed control strategies based on theoretical grounds, mainly around the concept of specificity. The general idea is that a TMS effect is thought to be ‘neural’ when it is specific to a stimulation site, time point, or other parameters. The implicit assumption is that the non-neural effects of a TMS pulse lack such specificity and therefore do not produce differences between conditions.

Recently, we have challenged this assumption on empirical grounds [9]. We investigated the non-neural effects of pre-stimulus TMS on target detection and revealed that the clicking sound of a sham TMS coil systematically influences task performance. First, we showed that a TMS pulse acts as a warning signal that facilitates reaction times to subsequent visual stimuli. Second, we found that a lateralized TMS pulse causes an automatic shift of spatial attention to the corresponding side of space. Importantly, the discovery of these non-neural effects of TMS is not per se problematic; they rather help deciding what constitutes a valid control condition. In the context of target detection, these findings demonstrated that comparing different stimulation sites or time points can be insufficient because these strategies might fail to dissociate neural from non-neural effects. Being aware of such effects, however, it is obviously possible to design experiments that control for these factors.

In the present study, we set out to further develop the empirical basis of control conditions in TMS research. To this end, we applied not only sham TMS, but now also real TMS over the vertex at not only pre-, but now also post-stimulus time windows. Moreover, aside from the sensory target detection task, we now also added a more cognitive ‘angle judgment’ task. We address three questions that have direct relevance for common control strategies in TMS research.

First, are there time-specific non-neural effects of single TMS pulses? By measuring a broad range of TMS time windows we aimed to replicate previously reported facilitatory non-neural effects of pre-stimulus TMS [9] and identify potentially specific non-neural effects of post-stimulus TMS. We hypothesized that post-stimulus TMS impairs task performance: the mere presence of the clicking sound of the TMS coil during each trial might create an expectancy that is violated when the TMS pulse occurs relatively late. This could result in delayed responses because participants ‘wait’ for the TMS pulse [10].

Second, are the non-neural effects of TMS dependent on experimental task? Despite our previous findings in the context of target detection reported above, one could argue that others tasks that involve higher cognitive functions and require a more complex stimulus response mapping are immune to such effects. For that reason, we assessed the non-neural effects of TMS on two tasks, namely a target detection task and an angle judgment task. This comparison provides information about the generalizability of the non-neural effects of TMS and, importantly, also has direct implications for TMS experiments that employ control tasks in order to demonstrate specificity.

Third, are the non-neural effects of sham TMS comparable to the non-neural effects of real TMS? This question is particularly relevant for experiments predominantly relying on sham TMS to control for non-neural effects. We compared the effects of real and sham TMS over the vertex. Assuming that real TMS over the vertex does not produce any neural effects, this comparison allows assessing the effect of the clicking sound of the TMS coil on task performance and to dissociate this from the added influence of the somato-sensory effect of TMS.

## Materials and Methods

### Ethics Statement

The study was approved by the medical-ethical committee of the University Medical Center, Maastricht, the Netherlands. All participants gave written informed consent prior to participation and were screened for TMS experimentation safety by a medical supervisor.

### Participants

Fourteen participants (8 female, aged 19 to 28) were recruited from the student population of Maastricht University. All had normal or corrected-to-normal vision and were right handed. The research question and hypotheses remained unknown to the participants until the end of the experiment.

### Stimuli and Task

We investigated the neural and non-neural effects of TMS over vertex on two different tasks, namely a detection task and an angle judgment task (see below). Stimuli were presented on a gamma-corrected 17″ TFT screen (Samsung SyncMaster 931 DF) at 57 cm viewing distance with the head supported by a chin rest. The video mode was 1280 x 1024 at 60 Hz and background luminance was 25 cd/m^2^. The Presentation software package (NeuroBehavioural Systems, Albany, CA) was used to control stimulus presentation and recording of behavioral responses. For both tasks, responses were given on the numeric keypad of a standard keyboard.

**Figure 1:**
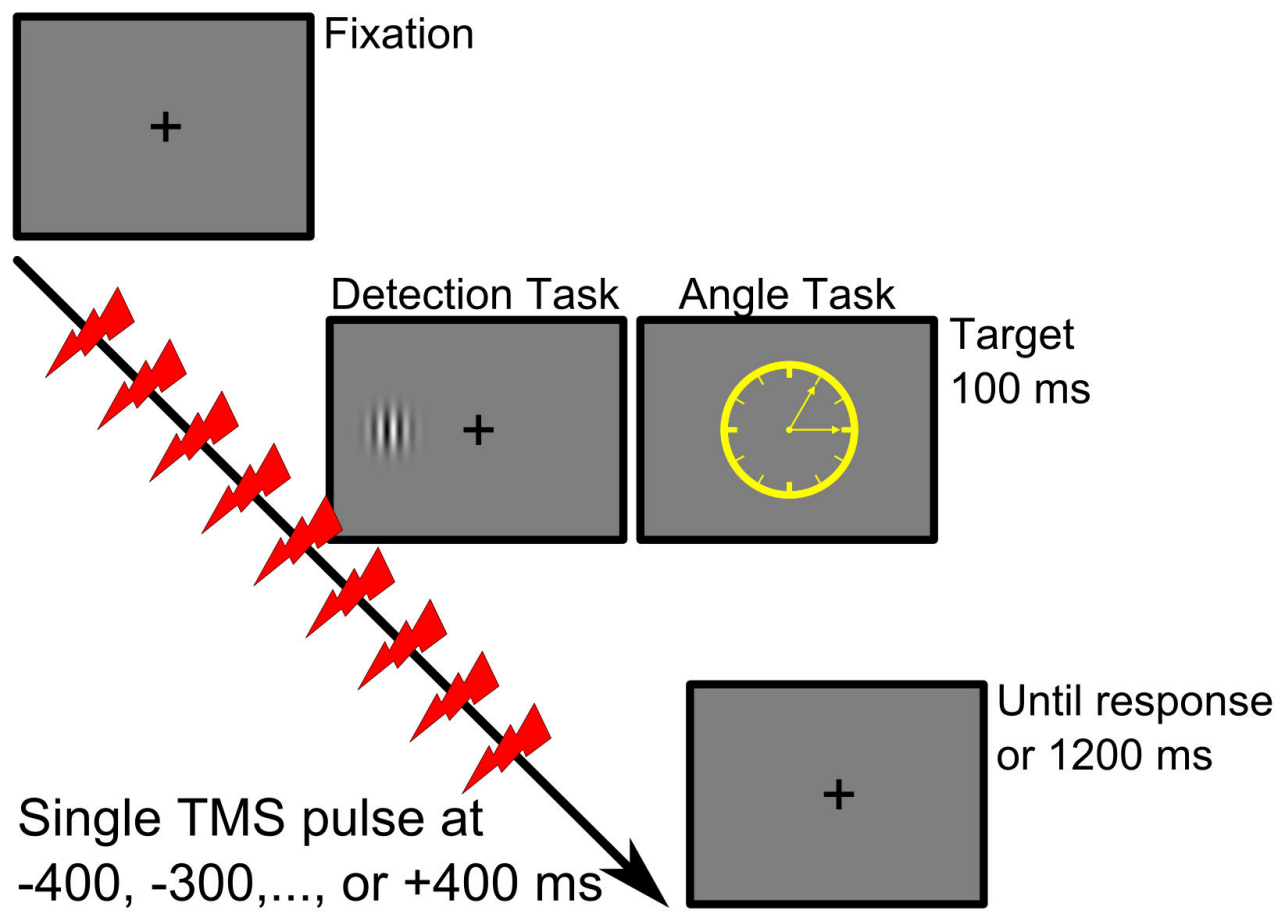
Example of a single trial for each task. Real or sham TMS was applied at one out of nine possible time points during each trial covering pre- and post-stimulus time windows ranging from -400 ms to + 400 ms relative to stimulus onset.

The detection task (Figure 1) required participants to perform a single button press whenever a target stimulus was presented irrespective of target location. A fixation cross was continuously presented at the centre of the screen and Gabor patches served as target stimuli that were shown at 400 ms after trial onset for 100 ms either left or right of the fixation cross at 7 degrees eccentricity (spatial frequency = 1.5 cycles per degree; envelope standard deviation = 0.75 degrees; Michelson contrast = 60%; random orientations). Participants were instructed to press the “NUM1” key with the right index finger as fast as possible as soon as the target stimulus appeared. The average inter-trial interval was four seconds with a jitter of ±500 ms.

The angle judgment task (Figure 1) required participants to judge the angle formed by two arrows similar to hour and minute hands on an analogue clock face [10–13]. On each trial, one out of twenty-four pre-generated clock faces with a diameter of 10 degrees of visual angle was presented at 400 ms after trial onset for 100 ms at the centre of the screen. The angle formed by the clock hands was 30, 60, 90, or 120 degrees and participants had to discriminate small (30 and 60 degrees) from large angles (90 and 120 degrees). Participants were instructed to press the “NUM1” key with their right index finger for small angles and the “NUM2” key with their right middle finger for large angles (90 and 120 degrees). A fixation cross was continuously presented between stimuli at the center of the screen. As above, the average inter-trial interval was four seconds with a jitter of ±500 ms.

### Procedure and Design

A within-subject design was employed in which all participants completed two TMS sessions on non-consecutive days. At the beginning of each session, participants received detailed instructions for the detection task and angle judgment task and completed 20 practice trials to get acquainted with the stimuli, timing, and required responses. During the main part of the experiment, participants performed blocks of both tasks in alternation, receiving real TMS in the first session and sham TMS in the second session, or vice versa (counterbalanced across participants). During all TMS trials, a single TMS pulse was delivered to the vertex at one out of nine possible TMS time windows (ranging from -400 to +400 ms in steps of 100 ms) time-locked to stimulus onset. The timing of the TMS pulse was task-irrelevant and provided no information about the properties of the stimulus. We also included trials without TMS that were interleaved with TMS trials. For both tasks, participants completed four blocks each consisting of 60 trials in randomized order resulting in 24 trials for each condition (including noTMS trials). Additionally, we included blocks of 24 trials without TMS at the beginning, halfway, and at the end of the experiment. Between blocks, participants could take a short break, were informed about which task was to come next, and whether or not TMS would be applied.

### Transcranial Magnetic Stimulation

Single biphasic TMS pulses were applied using a Medtronic MagPro X100 stimulator (Medtronic Functional Diagnostics A/S, Skovlunde, Denmark) and a figure-of-eight TMS coil (MC-B70; inner radius = 10 mm; outer radius = 50 mm). The stimulation intensity when applying real TMS was set at 50% maximum stimulator output for all participants. For the sham stimulation, a placebo TMS coil was used (MC-P-B70) with the same mechanical outline and clicking sound when discharging but equipped with a magnetic shield that reduces the effective magnetic field by approximately 80%. Here, the stimulation intensity was set at 30% maximum stimulator output in order to match the sound level produced by the real TMS coil. At this stimulation intensity, a sham TMS pulse is too weak to produce any neural effect and hardly any sensation on the head can be perceived, except for weak vibrations of the coil. Participant received real TMS and sham TMS over the vertex, that is electrode position Cz as defined by the International 10-20 system [14]. Using a mechanical arm, the TMS coil was placed on the head with the handle pointing posterior and the initial current direction going away from the handle.

### Statistical Analysis

The analysis of reaction times was performed after exclusion of all incorrect trials and outliers were removed from the data using the 1.5 x IQR (interquartile range) criterion. We then submitted individual mean reaction times and accuracies to repeated measures ANOVAs. Depending on the hypothesis being tested, within-subject factors were TMS time window (9 levels: -400, -300, -200, -100, 0, 100, 200, 300, 400 ms), TMS type (2 levels: real, sham), task (2 levels: detection, angle judgment), or context of no TMS trials (2 levels: interleaved, blocked). In cases where the sphericity assumption was violated, we performed a Greenhouse-Geisser correction but for simplicity the degrees of freedom are reported uncorrected. Effects of interest were explored with paired t-tests with Bonferroni correction for multiple comparisons unless stated otherwise.

## Results

### Difference between Blocked and Interleaved noTMS Trials

We collected data in the absence of TMS either presented interleaved with TMS trials or as a separate block. It has previously been shown that infrequent interleaved noTMS trials can have oddball-like properties simply by being in a context where TMS pulses are administered during the majority of trials [9]. This can cause unusual slowing of reaction times and, in the context of the present study, would invalidate their use as baseline condition. In line this view, a repeated-measures ANOVA on reaction time during noTMS trials with task (detection, angle judgment), TMS type (sham, real), and context (interleaved, blocked) as within subject factors revealed a highly significant effect of context (*F*_(1,13)_ = 59.940, *p* < .001) and an effect of task (*F*_(1,13)_ = 379.706, *p* < .001) due to differences in task difficulty. The main effect of context resulted from faster reaction times during blocked noTMS trials compared to interleaved noTMS trials (mean difference = 22 ms). This indicates that the mere presence of TMS trials creates a context that also modulates performance on interleaved trials without TMS. Consequently, we used blocked noTMS trials as baseline for subsequent analyses instead.

### Non-Neural Effect of TMS on Accuracy

A repeated-measures ANOVA on accuracy with task (detection, clock), TMS type (real, sham), and TMS time window (-400 ms, -300 ms …, +400 ms) as within subject factors revealed a significant three-way interaction (*F*_(8,104)_ = 3.867, *p* = .008). Somewhat surprisingly, this already shows that non-neural effects of single TMS pulses depend on a complex interplay between various experimental factors, including nature of the experimental task and pulse timing, and that they are not equivalent for sham and real TMS. This interaction was further explored by analyzing the data for each task separately.

**Figure 2:**
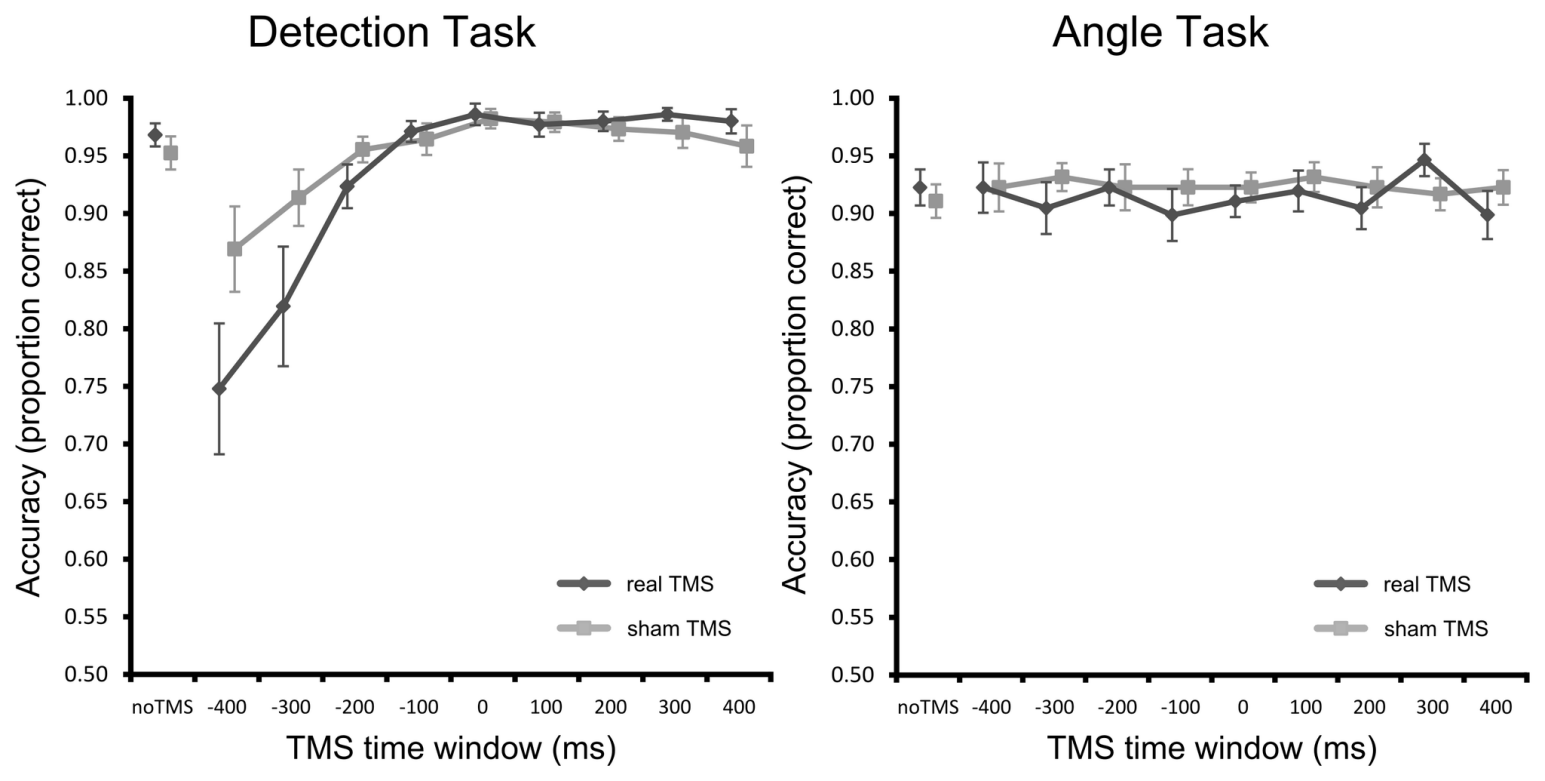
The effect of real and sham TMS on accuracy. For the detection task, decreased accuracy was observed at pre-stimulus TMS time windows with effects being stronger for real TMS compared to sham TMS. For the angle task, no effect of TMS was found on accuracy. Data points are slightly shifted sideways for clarity. Error bars show the standard error of the mean.

For the detection task, a repeated-measures ANOVA on accuracy with TMS type and TMS time window as within subject factors revealed a significant main effect of TMS time window (*F*_(8,104)_ = 12.002, *p* = .001), no effect of TMS type (*F*_(1,13)_ = 3.37, *p* = .090), and a significant interaction (*F*_(8,104)_ = 5.478, *p* = .008). The main effect of TMS time window resulted from decreased accuracy (due to false alarms) during the earliest TMS time windows, when the TMS pulse preceded the target stimulus, which then gradually increased and leveled off when the TMS pulse temporally coincided with target appearance. However, as indicated by the interaction between TMS type and TMS time window, this impairment in task performance differed between real TMS and sham TMS (Figure 2). Post-hoc paired-samples t-tests (uncorrected) showed that the effect on accuracy was more pronounced when applying real TMS compared to sham TMS. This effect was strongest at 400 ms prior to target appearance (*t*_(13)_ = 2.986, *p* = .011) but a trend was still visible at -300 ms (*t*_(13)_ = 1.978, *p* = .069) and -200 ms (*t*_(13)_ = 1.759, *p* = .102). No significant differences were observed for all remaining TMS time windows (all *p* > .10).

For the angle judgment task, a repeated-measures ANOVA on accuracy with TMS type and TMS time window as within subject factors did not reveal any significant effects. Neither TMS type (*F*_(1,13)_ = .863, *p* = .370) nor TMS time window (*F*_(8,104)_ = .548, *p* = .753) had an effect on accuracy and also the interaction was far from being significant (*F*_(8,104)_ = .785, *p* = .617).

In summary, we found that TMS only had a non-neural effect on accuracy on the detection task whereas the angle judgment task was unaffected. Moreover, target detection was specifically impaired at pre-stimulus time windows with effects being stronger when applying real TMS compared to sham TMS. These effects were due to increased false alarm rates, reflecting failure to withhold an already prepared response when the TMS pulse was given.

### Non-Neural Effect of TMS on Reaction Time

**Figure 3:**
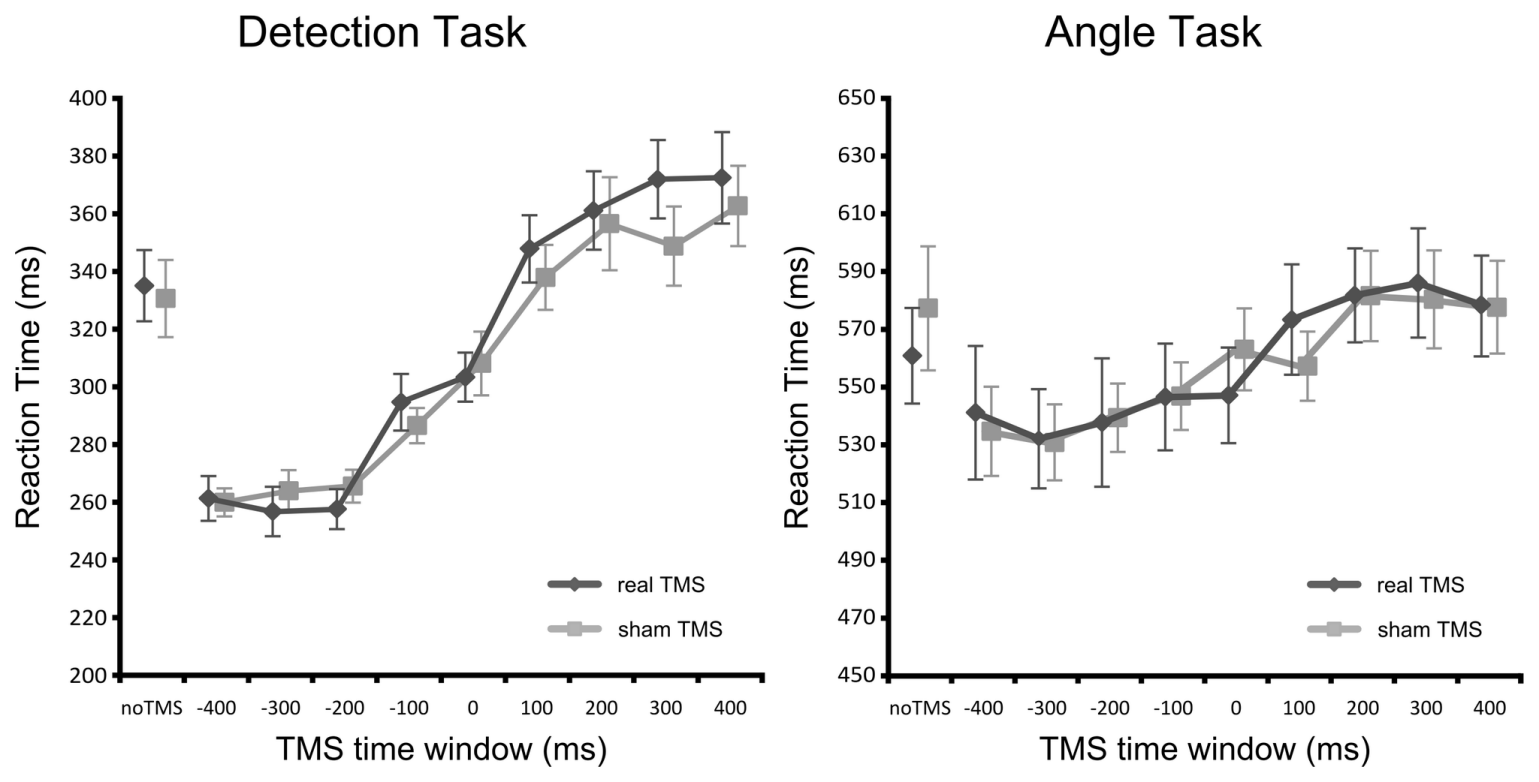
The effect of real and sham TMS on reaction times. For both tasks, reaction times were fastest at pre-stimulus TMS time windows and gradually increased with modulations still visible at post-stimulus TMS time windows. There was no difference between real and sham TMS. Data points are slightly shifted sideways for clarity. Error bars show the standard error of the mean.

A repeated-measures ANOVA on reaction time with task (detection, clock), TMS type (real, sham), and TMS time window (-400 ms, -300 ms …, +400 ms) as within subject factors revealed significant main effects of task (*F*_(1,13)_ = 520.489, *p* < .001) and TMS time window (*F*_(8,104)_ = 47.010, *p* < .001). Moreover, we found a highly significant interaction between task and TMS time window (*F*_(8,104)_ = 20.740, *p* < .001). The factor TMS type did not influence reaction time, neither the main effect nor any interaction with this factor reached significance (all *p* > .10). See Figure 3 for a complete overview of the data.

The main effect of task resulted from faster reaction times on the detection task compared to the clock task. This was simply due to differences in task difficulty because the detection task only required a button press after target appearance whereas the clock task involved angle judgments and two response alternatives.

**Figure 4:**
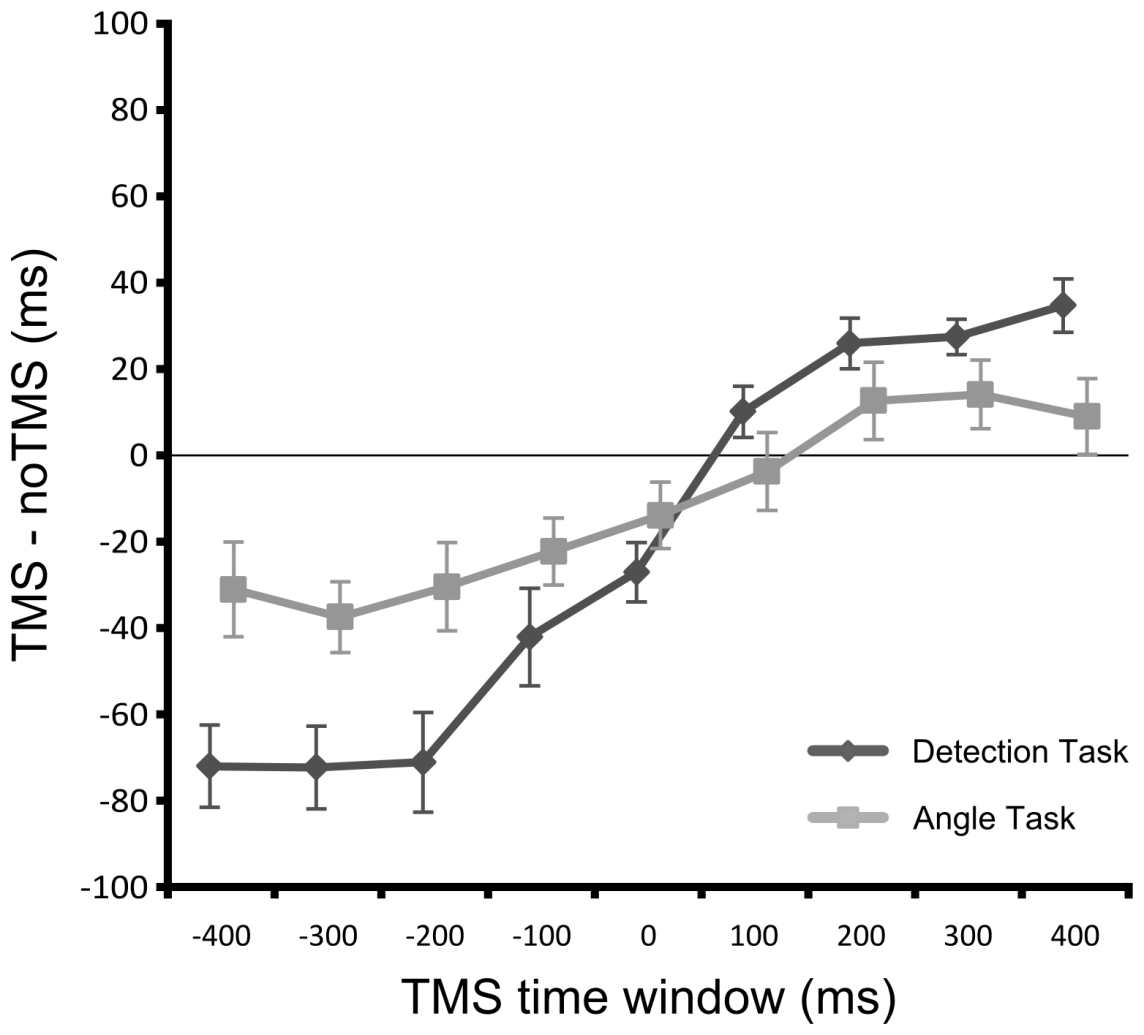
Time- and task-dependent effect of TMS on reaction times. For both tasks, reaction times were improved at pre-stimulus TMS time windows and impaired at post-stimulus TMS time windows. This effect was more pronounced at pre-stimulus TMS time windows during the detection task compared to the angle task but no significant difference between tasks was observed at post-stimulus TMS time windows. Data points are slightly shifted sideways for clarity. Error bars show the standard error of the mean.

The main effect of TMS time window resulted from gradually increasing reaction times across TMS time windows. Performance was best when the TMS pulse preceded target appearance and got worse the later the TMS pulse was applied with modulations still being visible for post-stimulus TMS time windows. However, as indicated by the interaction between task and TMS time window, this time-dependence of task performance was not the same for both tasks. Consequently, we directly compared both tasks to examine this differential effect of TMS time window after subtracting the individual baseline for each participant (in order to remove global task effects). Additionally, we collapsed the data across the factor TMS type because this factor did not influence task performance. As above, this analysis revealed a highly significant interaction between task and TMS time window (*F*_(8,104)_ = 20.703, *p* < .001) with TMS effects being more pronounced at pre-stimulus time windows in the detection task compared to the angle judgment task (Figure 4). Paired-samples *t*-tests (uncorrected) statistically confirmed this effect, showing significant differences between tasks for early pre-stimulus TMS time windows at -400 ms (*t*_(13)_ = 3.399, *p* = .005), -300 ms (*t*_(13)_ = 2.975, *p* = .011), and -200 ms (*t*_(13)_ = 2.863, *p* = .013). No significant differences were observed for all remaining TMS time windows (all *p* > .15) but there was a marginally significant effect at +400 ms (*t*_(13)_ = 2.057, *p* = .060).

In summary, we found that sham TMS and real TMS had the same effect on reaction times. Performance was modulated in a time-dependent way characterized by improvements at pre-stimulus TMS time windows and impairments at post-stimulus TMS time windows. Moreover, the detection task was more vulnerable to these modulations than the angle judgment task which showed the same pattern but to a lesser extent.

## Discussion

It is often assumed that the non-neural side effects of TMS such as the clicking sound of the TMS pulse are non-specific, that is, they do not influence task performance dependent on TMS parameters. However, we have recently challenged this assumption on empirical grounds [9]. Given that a valid interpretation of TMS results heavily relies on the adequacy of control conditions, and the fact that there are a range of controls to choose from (e.g. [15]), we believe that it is essential to gather empirical knowledge of the non-neural effects of TMS. Here, we further developed the empirical basis of control strategies in TMS research. Specifically, we addressed the time-dependence and task-dependence of the non-neural effects of TMS and compared real and sham TMS over vertex.

The most important finding of the present study is that the non-neural effects of TMS depend on a complex interplay between pulse timing, experimental task, and real or sham TMS. For both outcome measures, we found remarkably specific effects of these factors that interacted in meaningful ways. In the absence of appropriate control conditions, such effects could easily be falsely attributed to the neural consequences of TMS. Our results caution against over-simplistic assumptions regarding the non-neural effects of TMS but also help designing well-controlled TMS experiments.

### Time-Dependence of Non-Neural Effects of TMS

Previous work has shown that the clicking sound of a TMS pulse can facilitate target detection when applied prior to or in close temporal proximity of a target stimulus, primarily by acting as a warning signal and due to inter-sensory facilitation, respectively [5–9,16,17]. Here, we investigated the non-neural effects of TMS for a broad range of TMS time windows including pre- and post-stimulus stimulation. To begin with, our results replicate earlier findings of pre-stimulus facilitation. A TMS pulse that is administered prior to a stimulus provides temporal information and, consequently, increases the readiness to respond resulting in decreased reaction times. Additionally, we found impaired task performance at post-stimulus time windows. Reaction times were considerably prolonged when the TMS pulse occurred late during a trial; probably due to expectancy violations that caused participants to ‘wait’ for the TMS pulse (as suggested, but not systematically investigated, by [10]).

Reaction times were not the only outcome measure that revealed a time-dependent modulation of task performance. For the early pre-stimulus TMS time windows, we also found decreased accuracy on the detection task due to higher rates of false alarms. Apparently, participants were sometimes prone to press the response key when a TMS pulse was administered even though the task required them to only react when the target stimulus appeared. Such a failure to withhold an already prepared response until the proper ‘go’-signal is presented is well known from the response inhibition literature (for a review, see 18). As expected, this non-neural effect of TMS on accuracy was specific to the detection task because the clock task included two response alternatives so that response preparation could only occur once the stimulus was shown.

Taken together, this shows that there are time-dependent non-neural effects of TMS on reaction time and accuracy. Reaction times during pre- and post-stimulus TMS time windows were differentially affected by the clicking sound of the TMS coil leading to facilitation and impairment of task performance, respectively. This pattern of results was observed for the detection task as well as the clock task. In contrast, accuracies were only affected during pre-stimulus TMS time windows when target detection had to be reported by a speeded response with only one response alternative. A similar observation has recently been reported by Jacobs et al. [19] in the context of behavioral priming and masking. When applying TMS over the vertex as a control condition, they found time-dependent modulations of prime effectiveness with stronger effects at pre-stimulus TMS time windows. Interestingly, these effects were specific to the priming task whereas no time-dependent effects were observed on masking. Moreover, these differential effects of TMS were only found with TMS over the vertex but absent during sham TMS over early visual cortex. These findings therefore also have a bearing on the task-dependence of the non-neural effects of TMS and the differences between real and sham TMS which are discussed below.

Importantly, these results demonstrate that a comparison between different TMS time windows can fail to control for the non-neural effects of TMS in the context of a TMS experiment. As a consequence, we recommend using control strategies that provide control conditions within each TMS time window and to avoid comparisons across time windows of stimulation.

### Task-Dependence of Non-Neural Effects of TMS

One could argue that task performance on a simple detection task is particularly prone to be affected by the non-neural effects of TMS leaving the question in how far such results can be generalized. Other tasks that involve higher cognitive processes and require a more complex stimulus response mapping might be immune to such effects. Our results clearly show that the time-dependent effects on reaction time described above are qualitatively similar for the detection task and clock task even though the clock task consisted of a complex set of stimuli and required participants to (1) make an angle judgment (2) apply a decision rule based on this judgment, and (3) select the correct response alternative. It therefore seems highly plausible that similar non-neural effects of TMS can be observed across a broad range of tasks. Admittedly, both tasks used in the present study were reaction time tasks and accuracy on the clock task was unaffected by TMS. But, at the very least, our results show that the non-neural effects of TMS cannot be explained away as a low-level phenomenon with minimal scope.

While the presence of non-neural effects of TMS during both tasks is highly relevant for the generalizability of our findings, the differences between tasks are equally important. Interestingly, especially pre-stimulus effects on reaction times were more pronounced during the detection task compared to the clock task. Moreover, changes in accuracy were only observed for pre-stimulus TMS time windows during the detection task whereas the clock task was unaffected by TMS across all time windows. These differences between tasks have direct implications for the use of a control task as a control strategy in TMS experiments. A control task is one of the many possible approaches to demonstrate that a TMS effect is indeed ‘neural’. The idea is that if stimulation of a brain area only affects performance on task ‘A’ but not task ‘B’ then the brain area in question is only functionally relevant for that particular task [15]. However, in case the non-neural effects of TMS also have a differential effect on the two tasks this reasoning obviously fails. Our results show that this can indeed be the case and a control task therefore has to be well-matched. Otherwise differences in the non-neural effects of TMS between tasks can be misinterpreted and produce false positive or false negative findings.

### Real versus Sham TMS

We also compared the effect of real and sham TMS over the vertex. For the most part, we did not find significant differences between these two conditions with all effects on reaction time being identical. However, the drop in accuracy for pre-stimulus TMS time windows during the detection task was more pronounced when applying real TMS compared to sham TMS. While a neural explanation of this effect cannot be excluded, we propose that this increased rate of false alarms was due to somato-sensory effects. In the present study, a sham TMS coil was used that produced the same clicking sound as real TMS but almost completely lacked the somato-sensory effects that are typically associated with a TMS pulse. In our view, the combined auditory and somato-sensory effects act as a stronger trigger to press the response key than the clicking sound of the TMS coil in isolation. Our results therefore demonstrate that common sham TMS coils are an approximation of the non-neural effects of real TMS but are not perfect. They fail to mimic some non-neural aspects of TMS and, consequently, cannot control for them. Importantly, the somato-sensory effects during vertex stimulation are mild whereas other stimulation sites can cause stronger sensations and even discomfort. Under such circumstances, the differences between sham and real TMS become larger and most likely more problematic. For example, it has been found that subjective ratings of unpleasantness of TMS were negatively correlated with performance on a working memory task [20]. However, the development of more advanced sham TMS coils is certainly possible and our results do not invalidate the use of existing sham TMS coils.

A widely used control strategy is to apply TMS over the vertex. It is assumed that this stimulation site has no neural effects and therefore qualifies as a baseline and control condition. As pointed out above, there were almost no differences between sham and real TMS except for the slightly stronger drop in accuracy at pre-stimulus TMS time windows during the detection task. On the one hand, these results indicate that real TMS over the vertex has advantages over sham TMS as a control condition because it accounts for mild somato-sensory effects of TMS without producing unintended neural effects on task performance. On the other hand, this advantage comes at the cost of reduced flexibility in TMS coil positioning. One of the strongest arguments in favor of sham TMS is that it can be applied to any brain area and is therefore better suited to control for stimulation site-dependent non-neural effects of TMS. For example, we have previously shown that a lateralized sham TMS pulls covert spatial attention towards the corresponding side of space thereby facilitating target detection in this hemifield [9]. Such effects can hardly be controlled for with vertex stimulation. This illustrates that there is no perfect control strategy yet and, as a consequence, it is necessary to evaluate the pros and cons of each approach in the context of the specific research question at hand.

## Conclusions

We provide novel evidence regarding the time-dependence and task-dependence of the non-neural effects of TMS and assessed the differences between real and sham TMS over vertex. Critically, we show that non-neural TMS effects depend on a complex interplay of these factors. Knowledge of such effects is of critical importance for the interpretation of TMS experiments and helps deciding what constitutes an appropriate control condition. Our results broaden the empirical basis of control strategies in TMS research and point at potential pitfalls that should be avoided. Otherwise, the non-neural effects of TMS can be falsely attributed to the intended TMS manipulation.
